# Predators do not spill over from forest fragments to maize fields in a landscape mosaic in central Argentina

**DOI:** 10.1002/ece3.3247

**Published:** 2017-08-22

**Authors:** Marco Ferrante, Ezequiel González, Gábor L. Lövei

**Affiliations:** ^1^ Department of Agroecology Flakkebjerg Research Centre Aarhus University Slagelse Denmark; ^2^ Centro de Investigaciones Entomológicas de Córdoba Instituto Multidisciplinario de Biología Vegetal Universidad Nacional de Córdoba, CONICET Córdoba Argentina

**Keywords:** biological control, chaco serrano, ecosystem services, edge effect, fragmentation, sentinel prey

## Abstract

South America is undergoing a rapid and large‐scale conversion of natural habitats to cultivated land. Ecosystem services still remain important but their level and sustainability are not known. We quantified predation intensity in an Argentinian agricultural landscape containing remnants of the original chaco serrano forest using artificial sentinel prey. We sought to identify the main predators and the effect of landscape configuration and maize phenology on predation pressure by invertebrate and vertebrate predators in this landscape. The most common predators were chewing insects (50.4% predation events), birds (22.7%), and ants (17.5%). Overall predation rates in forest fragments (41.6% per day) were significantly higher than in the surrounding maize fields (21.5% per day). Invertebrate predation was higher inside and at the edge of forest fragments than within fields, and did not change with increasing distance from a fragment edge, indicating a lack of spillover from the native habitat remnants to the cultivated matrix at the local scale. Distance from a continuous forest had a positive impact on predation by invertebrates and a negative impact on vertebrate predation.

## 1 INTRODUCTION

With the increasing size of the human population, demand on various resources has accelerated dramatically (Steffen, Broadgate, Deutsch, Gaffney, & Ludwig, 2015). This “Great Acceleration” has impacts on large‐scale ecological processes that form the basis of ecosystem services (ESs; de Groot, Wilson, & Boumans, 2002), on which sustainable agriculture depends (Tilman, Cassman, Matson, Naylor, & Polasky, 2002). Because biodiversity provides ESs, the two concepts are not always separated (Mace, Norris, & Fitter, 2012), although neither of them should be used as proxy for the other. Current agricultural production faces a serious challenge due to its dependence on massive non‐renewable external inputs (Gliessman, 2015). Increased reliance on ESs for sustainable agricultural production is inevitable. This situation brings up important challenges to: (1) quantify the intensity of, as well as track and directly monitor changes in ESs, (2) identify the effect of agricultural management practices on ESs, and (3) develop landscapes that sustain ESs (Tscharntke, Klein, Kruess, Steffan‐Dewenter, & Thies, 2005).

Conversion of natural areas to agriculture remains among the major drivers of biodiversity loss (Fahrig, 2003; Tscharntke et al., 2012). Such conversion generates landscapes consisting of a matrix of cultivated areas, in which natural habitat fragments varying in number, size, and distance from each other are embedded (Fahrig, 2003). Traditionally, the remaining natural habitat fragments were considered refuges not only for native biodiversity, but also for species providing pest control (Bianchi, Booij, & Tscharntke, 2006), or pollination (Kremen, Williams, Bugg, Fay, & Thorp, 2004); the matrix was seen unsuitable to sustain these populations (Simberloff & Abele, 1976). However, this turned out to be an oversimplification of a dynamic relationship between habitat fragments, edges, and matrix (Forman & Godron, 1981). Both the matrix (Kupfer, Malanson, & Franklin, 2006) and the edges (Forman & Baudry, 1984; Magura, Lövei, & Tóthmérész, 2017) have great influence on the communities within the fragments. Individuals frequently move between these landscape elements (Blitzer et al., 2012; González, Salvo, Defagó, & Valladares, 2016), and some species are closely related to the edges themselves (Duelli & Obrist, 2003; Lövei, Magura, Tóthmérész, & Ködöböcz, 2006).

Natural habitats often increase the diversity and abundance of natural enemies (Bianchi et al., 2006; Chaplin‐Kramer, O'Rourke, Blitzer, & Kremen, 2011). Area (Fahrig, 2003), isolation (Kruess & Tscharntke, 2000), permeability (i.e., perimeter/area ratio, (Stoner & Joern, 2004; Wu, 2007)), and proximity to noncrop habitats (Clough, Kruess, Kleijn, & Tscharntke, 2005; González, Salvo, & Valladares, 2015; Tscharntke, Gathmann, & Steffan‐Dewenter, 1998) influence arthropod densities and distribution, and their beneficial effects on crops. Temporal dynamics is also important, as movements of natural enemies between natural fragments and crops change in direction and intensity (Macfadyen et al., 2015; Rand, Tylianakis, & Tscharntke, 2006). Less is known about the effects of landscape structure on predation, particularly by generalist species (Chaplin‐Kramer et al., 2011).

Here, we examine the relationship between predation pressure and landscape parameters in a recently converted, cultivated landscape in central Argentina. The original vegetation was chaco serrano, one of the most threatened subtropical habitats, as 94% of its original area has been recently converted to large‐scale maize and soybean production (Zak, Cabido, & Hodgson, 2004). Earlier studies documented the biodiversity of the remaining fragments (González, Salvo, & Valladares, 2017a; González et al., 2015), and the movement of certain beneficial arthropods between forest remnants and the surrounding cultivated areas (González et al., 2016).

Specifically, we tested the following hypotheses:

H1: Predation pressure in forest fragments is higher than in their cultivated surroundings. We expected this because the forest fragments have higher primary production, larger standing biomass, and less disturbance than the crop, all of which can generate more food for herbivores, thus indirectly favoring predators, and cause higher predation pressure.
H2: Predation pressure at the edge is higher than either in the centre of the fragment, or in the matrix. Natural enemies residing in edges may benefit from complementary resources from both adjacent habitats (Ries, Fletcher, Battin, & Sisk, 2004), and reach higher densities or activities there. Additionally, the edge can support a specific set of edge‐preferring species (Duelli & Obrist, 2003), and the higher predator diversity may increase predation pressure.
H3: Predation pressure is higher in fragments which are larger or closer to the supposed source habitat, the not converted, continuous forest, than in smaller fragments, or in those farther away from these source habitats. In this landscape, larger fragments have higher densities of natural enemies (González et al., 2015), and flying natural enemies move out of the forest fragments more than into those (González et al., 2016). Moreover, predation pressure can be positively correlated with edge density or perimeter length, because there often are local density increases at edges (Andrén, 1995).
H4: Predation pressure by invertebrate predators decreases with increasing distance from the fragment edge, due to a decrease in densities or mobility of natural enemies that reside in the forest fragment but move out to feed in the surrounding crop (spillover or halo, Blitzer et al., 2012). While invertebrate predators can be affected by factors at small scales (Gaston & Blackburn, 1996), we did not expect such gradient for vertebrate predators that have higher mobility.
H5: Invertebrate predation pressure would be positively related to ground cover, because ground‐active arthropods prefer vegetation or litter against bare ground (Koivula, Punttila, Haila, & Niemelä, 1999; Magura, 2002) and have higher densities in such patches.
H6: Predation reaches its peak during maize flowering, as a consequence of increased predator densities at this time. This could happen either because these natural enemies consume pollen themselves or because they are attracted to the field by the increased density of other, pollen‐feeding arthropods (Pilcher, Rice, & Obrycki, 2005).

We found predation rates up to 42% per day, constituting strong top‐down effects in this landscape. There was qualified support for our hypotheses: invertebrate but not vertebrate predation rates were significantly higher within the forest fragments and along the edges than within the crop. Ground cover increased predation pressure but only in the maize fields. Contrary to expectations, distance from the continuous forest was positively related to invertebrate, while negatively to vertebrate predation pressure, indicating that vertebrate and invertebrate predators perceive the same landscape differently.

## 2 MATERIALS AND METHODS

### 2.1 Study site

Our study site was located in Córdoba Province (31.10°–31.30°S and 64.00°–64.30°W) in central Argentina. The original vegetation of the study area is chaco serrano, the southern part of a seasonally dry forest, gran chaco, with *Aspidosperma quebracho‐blanco* and *Schinopsis quebracho* forming the canopy, and a slightly lower subcanopy made up of several leguminous species. There is a scrub‐like shrub and herbaceous layer. Due to conversion mainly during the 20th century, chaco forest today is restricted to fragments of varying size in a cultivated landscape; the larger patches cover terrain unsuitable for large‐scale, mechanized agriculture typical of the region (Nanni & Grau, 2014). The dominant crop in the region is maize (*Zea mays*), an important crop in Argentina (planted on almost 5 million ha, FAOStat, 2017). In this landscape, we selected eleven forest fragments as different in size as possible (0.5–15 ha, Table S1). For each fragment, we took the following measurements from Google Earth Images (https://www.google.com/earth/):

1. Fragment size (ha), fragment perimeter length (m), edge density (“ED”) calculated as the ratio of fragment perimeter and area (Helzer & Jelinski, 1999);
2. Degree of isolation. Various measures of isolation were calculated: the shortest distance from the nearest neighboring forest fragment (“Isolation 1”) (Krebs, 1999), the shortest distance from the sampled edge of a fragment to the next (“Isolation 2”), the shortest distance between the given forest fragment and the nearest edge of the continuous, native forest (“Isolation 3”), and the shortest distance between the forest fragment and the continuous native forest by a “stepping stone” process of dispersal (“Isolation 4”, see example in Fig. S1). However, Isolation 3 and Isolation 4 were highly correlated, and in order to avoid multicollinearity, we only used the simplest measurement, Isolation 3, for the analysis.

### 2.2 Measuring predation

During the southern summer (January–March) of 2016, we measured predation intensity at eight positions at each fragment: in the interior (>15 m from the edge), at the edge (defined as the transitional, uncultivated area between the forest fragment and the maize field), and at 1, 2, 5, 10, 20, and 40 m from the edge into the maize field. We used artificial caterpillars (15 mm long, 3 mm diam.) made of green plasticine (Smeedi plus, V. nr. 776609, Denmark) (Howe, Lövei, & Nachman, 2009). To minimize the risk of damaging the caterpillars during handling, they were glued individually on small pieces of reed and transported to the field in glass tubes. At each position, we placed five caterpillars at 1 m distance from each other, giving a total of 40 caterpillars per site. Sentinel prey were placed in the shadow to avoid damage by direct sunlight in the morning and were left exposed to predators for 24 hr. The following day they were inspected in the field for signs of predation, using a hand‐held magnifying glass (20×). If necessary, caterpillars were transported to the laboratory for verification and photographing. Signs of predation were identified from photographs in published papers (Ferrante, Lo Cacciato, & Lövei, 2014; Low, Sam, McArthur, Posa, & Hochuli, 2014). Note that our method cannot distinguish whether such higher predation pressure would emerge from higher predator density, higher predator activity, or a combination of the two. There were six sampling sessions starting on 14 January 2016, when maize was ~16 cm tall (BBCH phenological stage 15–16, (Lancashire et al., 1991)), and ending on 28 March 2016, when maize was at milky ripening (development stage 89). In total, 2,600 artificial caterpillars were exposed, of which 30 (1.15%) were lost. The largest fragment only had five sessions (no prey exposed on 14 January 2016).

### 2.3 Habitat characterization

At every sampling location, we photographed two different areas on the soil surface, each of 25 cm × 50 cm, identified with the help of a metal frame. From these images, we calculated the area of bare ground (“BareGround”, in %), as well as the area covered by live (“LivePlant”, in %) and dead plant (“DryGround”, in %) material, using the program ImageJ. For evaluation, we used the mean values measured on the two photograph frames per position.

### 2.4 Data analysis

In order to test which landscape factors influence predation intensity, we used a multimodel information‐theoretic (IT) approach (Burnham & Anderson, 2003). The approach consists of specifying a set of candidate models based on *a priori* knowledge or specific hypotheses, ranking the models from the lowest to the highest AIC value (Akaike, 1998) and Aikake weight (AICw) (Burnham & Anderson, 2003), and averaging all the models with ΔAIC < 2 or AICw ≥ 0.9. Models which do not fit such criteria lack sufficient support and are discarded (Burnham & Anderson, 2003). The IT approach is suitable for complex analyses which include many models and compared to the traditional null‐hypothesis testing for the model variable, it has the advantage of evaluating the support for each model simultaneously, and reducing model uncertainty by averaging the most reliable models (Zuur, Ieno, Walker, Saveliev, & Smith, 2009). Before specifying the models, we graphically tested each numerical factor for outliers using boxplots and dot charts, and for collinearity between factors using the Variance Inflation Factor (Ieno & Zuur, 2015). We did not find outliers, but there was collinearity between Position and BareGround, and between Area, Perimeter and ED.

To systematically address our different hypotheses, we separately analyzed total predation, and predation attributed to invertebrates, vertebrates, chewing insects (excluding ants), ants, birds, and small mammals. Ninety‐three candidate models for total predation and each of the invertebrate predators, and 47 for each of the vertebrate ones were defined avoiding collinear factors. Each set included models with a single factor, all the possible additive models with two factors, and all the possible additive models with two factors plus maize phenology. Site was always considered a random factor, while phenology was a random factor only in models which did not include it already as a fixed factor. We did not include other models with interactions as we did not have any specific *a priori* hypothesis for them. From the set of models of vertebrate predators, we also excluded models including BareGround, as the dimensions of this parameter were too small to be relevant for them. When examining factors influencing predation by vertebrates, position was coded as “forest”, “edge”, and “maize field”, without considering different distances within the crop, which are likely to be too small for predators with high mobility. For each set, we identified the best models, and the estimates of these were averaged to obtain the final model (Burnham & Anderson, 2003). Tukey's post hoc *t* test was used to identify significant differences in predation intensity for categorical variables (Phenology and Position). The statistical analysis was performed with the statistical program R, version 3.3.1 (R Core Team, 2016). The generalized linear mixed models were created using the package “*lme4*” (Bates, Mächler, Bolker, & Walker, 2014), the supported models averaged using the package “*MuMIn*” (Barton, 2016), and the post hoc Tukey *t* test was performed using the package “*multcomp*” (Hothorn, Bretz, & Westfall, 2008).

## 3 RESULTS

### 3.1 Predation pressure

In total, 692 artificial caterpillars were attacked, giving an overall median predation rate of 27.0% per day (range = 21.8%–32.9% per day, *n* = 11, Table 1). Four predator groups were identified: chewing insects (50.4% of all predation), and ants (17.5%) as invertebrate predators; birds (22.7%), and small mammals (10.0%) as vertebrate predators. Unknown predators accounted for 1.7% of the artificial caterpillars attacked. Within the maize field, the highest predation was found at 40 m from the edge (median = 26.7% per day, range = 6.9%–36.7% per day, *n* = 11). Chewing insect predation was highest at 1 m (mean = 10.8% per day, *SD* = 6.4% per day, *n* = 11), ant predation at 40 m (mean = 3.4% per day, *SD* = 3.4% per day, *n* = 11), bird predation at 20 m (mean = 11.0% per day, *SD* = 5.5% per day, *n* = 11), and small mammal at 10 m from the forest edge (mean = 1.6% per day, *SD* = 2.4% per day, *n* = 11).

**Table 1:** The number of artificial caterpillars attacked by various predators at Rio Ceballos, Córdoba, Argentina, during the southern summer of 2015/2016

Position	No. of caterpillars exposed	No. of caterpillars attacked by				
		Chewing insects^a^	Ants	Birds	Mammals	Unknown predators
Forest	324	66	52	1	21	2
Edge	322	86	22	5	34	0
1 m from edge	322	35	10	17	3	4
2 m from edge	319	26	8	27	3	2
5 m from edge	323	42	9	20	0	2
10 m from edge	319	34	3	21	5	2
20 m from edge	324	26	6	36	2	0
40 m from edge	317	34	11	30	1	0
Total	2,570	349	121	157	69	12

Caterpillars were placed at various positions in forest fragments and the surrounding maize fields. Multiple attacks by the same predator were counted as single attack, but attacks by different predators were considered independent.

a Excluding ants.

Except for total invertebrate predation, all predator groups had more than one model with the lowest AIC values, indicating the need for model averaging (Table 2). Two or three variables in each model were important to explain the observed trends in predation pressure, with maize phenology as the most frequent factor, while isolation measures and habitat were also relevant for most predators.

**Table 2:** A list of the best models for explaining predation rates by various predator groups at Rio Ceballos, Córdoba, Argentina, during the southern summer of 2015/2016, based on ΔAIC and model weight

Predator group	Best models	AIC	ΔAIC	*df*	Model weight
All predators	Isolation1 + Phenology + Distance + (Site)^a^	2,841.6	0.0	15	0.3770
	Isolation1 + Phenology + LivePlant + (Site)	2,842.5	0.8	9	0.2494
Invertebrates	Isolation3 + Phenology + Distance + (Site)	2,209.3	0.0	15	0.8683
Chewing insects^b^	Isolation3 + Phenology + LivePlant + (Site)	1,867.1	0.0	9	0.4497
	Area + Phenology + LivePlant + (Site)	1,868.4	1.3	9	0.2384
	Perimeter + Phenology + LivePlant + (Site)	1,869.8	2.7	9	0.1154
Ants	Isolation1 + Phenology + Distance + (Site)	890.5	0.0	15	0.2329
	Distance + Phenology + (Site)	891.1	0.6	15	0.1761
	Isolation3 + Phenology + Distance + (Site)	892.0	1.4	15	0.1143
	Isolation2 + Phenology + Distance + (Site)	892.1	1.6	15	0.1054
	Area + Phenology + Distance + (Site)	892.5	1.9	15	0.0897
Vertebrates	Isolation3 + Phenology + Isolation1 + (1|Site)	1,500.1	0.0	9	0.7961
	Isolation3 + Isolation1 + (Phenology) + (Site)	1,504.0	3.9	5	0.1143
Birds	Habitat + Phenology + Isolation1 + (Site)	1,104.2	0.0	10	0.6226
	Habitat + Phenology + (Site)	1,107.6	3.4	9	0.1162
	Habitat + Phenology + Isolation3 + (Site)	1,107.6	3.4	10	0.1133
Mammals	Area + Habitat + (Phenology) + (Site)	524.3	0.0	6	0.7219
	Habitat + Phenology + Isolation2 + (Site)	527.6	3.3	10	0.1373
	Habitat + Phenology + Area + (Site)	528.4	4.1	10	0.0923

a Factors in parenthesis are considered random factors.

b Excluding ants.

H1: Predation pressure in forest fragments higher than in cultivated habitats

Total predation rates within forest fragments (mean = 41.6% per day, *SD* = 12.5% per day, *n* = 11) were significantly higher (Tukey's *t* test, *p* < .001 for all comparisons) than in the maize fields, at any distance from the forest (mean = 21.5% per day, *SD* = 3.2% per day, *n* = 11).

The same was found for invertebrate predation rates (mean_forest_ = 34.7% per day, *SD* = 15.3% per day, *n* = 11; mean_crop_ = 12.6% per day, *SD* = 3.6% per day, *n* = 11; Tukey's *t* test, *p* < .001 for all comparisons), as well as for ant predation (mean_forest_ = 34.4% per day, *SD* = 15.0% per day, *n* = 11; mean_crop_ = 12.6% per day, *SD* = 3.6% per day, *n* = 11; Tukey's *t* test, *p* < .001 for all comparisons). Chewing insect predation was not significantly affected by the habitat.

Vertebrate predation within the forest fragments (mean = 6.9% per day, *SD* = 6.5% per day, *n* = 11) was not significantly different than within the crop (mean = 8.6% per day, *SD* = 3.3% per day, *n* = 11). Bird predation within the forest fragments (mean = 0.3% per day, *SD* = 1.0% per day, *n* = 11) was significantly lower (Tukey's *t* test, *p* < .01) than within the crop (mean = 7.9% per day, *SD* = 3.0% per day, *n* = 11), while the opposite was registered for mammal predation (mean_forest_ = 6.6% per day, *SD* = 6.0% per day, *n* = 11, mean_crop_ = 0.74% per day, *SD* = 0.79% per day, *n* = 11; Tukey's *t* test, *p* < .001).

H2: Predation pressure along forest edges higher than in the cultivated matrix or the centre of the fragment

**Figure 1:**
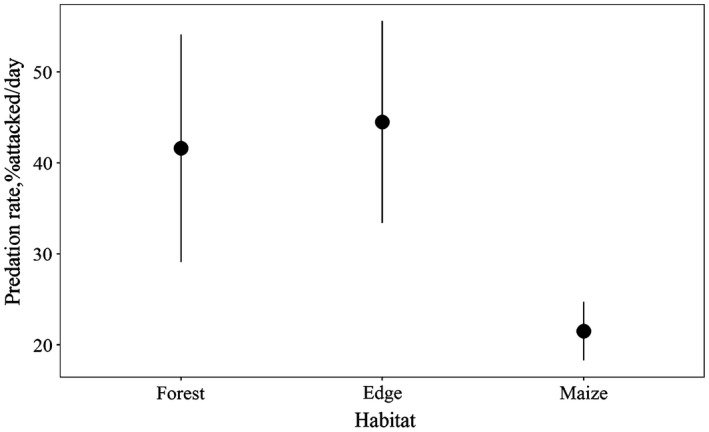
Mean daily predation (%±*SD*) at the 11 sites, within the forest fragments, along the edges, and within the maize field, at Rio Ceballos, Córdoba, Argentina, during the southern summer of 2015/2016

Overall predation rates along the edges of forest fragments (mean = 44.5% per day, *SD* = 11.1% per day, *n* = 11) were not higher than within forest fragments, but were significantly higher (Tukey's *t* test, *p* < .001 for all comparisons) than within the crop (mean = 21.5% per day, *SD* = 3.2% per day, *n* = 11, Figure 1).

Similarly, invertebrate predation along edges (mean = 33.1% per day, *SD* = 11.6% per day, *n* = 11) was not significantly different than within the forest fragments, but was significantly higher (Tukey's *t* test, *p* < .001 for all comparisons) than within the crop (mean = 12.6% per day, *SD* = 3.6% per day, *n* = 11). Ant predation at edges (mean = 33.1% per day, *SD* = 11.6% per day, *n* = 11) was significantly higher (Tukey's *t* test, *p* < .05) than predation at 10 m from the forest edge (mean = 0.9% per day, *SD* = 2.2% per day, *n* = 11), but not at other distances. Chewing insect predation was not significantly affected.

Vertebrate predation along forest edges (mean = 14.8% per day, *SD* =12.8% per day, *n* = 11) was not significantly higher than in the crop (mean = 8.6% per day, *SD* = 3.3% per day, *n* = 11), or within the forest fragments (mean = 6.9% per day, *SD* = 6.5% per day, *n* = 11). Bird predation rate along forest edges (mean = 1.5% per day, *SD* = 3.1% per day, *n* = 11) was not significantly different from predation inside the forest fragments (mean = 0.3% per day, *SD* = 1.0% per day, *n* = 11) but significantly lower (Tukey's *t* test, *p* < .001) than in the crop (mean = 7.8% per day, *SD* = 3.0% per day, *n* = 11). Mammal predation at edges (mean = 10.7% per day, *SD* = 12.4% per day, *n* = 11) was significantly higher (Tukey's *t* test, *p* < .001) than in the crop (mean = 0.74% per day, *SD* = 0.79% per day, *n* = 11) but not higher than in the fragments (mean = 6.6% per day, *SD* = 6.0% per day, *n* = 11).

H3: Predation pressure higher in larger fragments or closer to the source habitat

Fragment area had a significantly positive effect only on mammal predation (GLMM, *z* = 4.78, *p* < .001). On predation by chewing insects, contrary to the hypothesis, it had a significant negative effect (GLMM, *z* = 2.56, *p* < .05).

Distance from the closest neighboring fragment (Isolation 1) had a significant positive effect on total (GLMM, *z* = 2.49, *p* < .05), vertebrate (GLMM, *z* = 3.36, *p* < .001), bird (GLMM, *z* = 2.77, *p* < .01) predation rates, and a marginally positive effect on predation by ants (GLMM, *z* = 1.68, *p* < .1).

Distance from the closest neighboring fragment at the sampled edge (Isolation 2) had a significant negative effect on mammal predation (GLMM, *z* = 4.73, *p* < .001).

Distance from the continuous forest (Isolation 3) had a significant positive effect on predation by all invertebrates (GLMM, *z* = 3.62, *p* < .001), and chewing insects (GLMM, *z* = 2.79, *p* < .01), but a negative effect on vertebrate predation (GLMM, *z* = 4.66, *p* < .001).

Edge density did not affect predation by any group, while fragment perimeter had a significant negative effect on predation by chewing insects (GLMM, *z* = 2.16, *p* < .05).

H4: Predation pressure by invertebrate predators decreases away from the forest edge

Distance from the forest edge did not significantly affect predation rates.

H5: Predation pressure by invertebrate predators positively related to ground cover

Total and chewing insect predation rates were significantly (GLMM, *z* = 9.97, *p* < .001 and *z* = 8.77, *p* < .001, respectively) positively related to live plant cover, but the same was not true for invertebrate and ant predation rates. Other elements of surface cover (amount of dead plant material or bare ground) had no influence on predation rates by any group.

H6: Predation pressure peaks during maize flowering

Phenology had a significant influence on predation rates by all identified predator groups but mammals (Table 3). Total predation during the early milky ripening stage (mean = 37.8% per day, *SD* = 9.0% per day, *n* = 11) was significantly higher than any other phases (Tukey's *t* test, *p* < .001–.05) excluding maize ripening, which was only marginally significant (Tukey's *t* test, *p* < .1).

**Table 3:** Effects of the landscape variables on the seven final averaged models

	Total predation	Invertebrates	Chewing insects	Ants	Vertebrates	Birds	Mammals
Area		↓*	↓*	+			↑***
Isolation1	↑*			↑*	↑**	↑**	
Isolation2				+	↓*		
Isolation3		↑**	↑**	+	↓***		
Phenology	***	***	***	*	***	***	
Distance	***	***		***			
Habitat					*	**	***
LivePlant	↑***		↑***				

Arrows indicate positive (↑) or negative (↓) effect of a numerical variable, while symbols indicate significance levels (+*p* < .1; *p < .05; **p < .01; ***p < .001). Only variables with at least one significant value are shown. Edge density, fragment perimeter, % coverage by dead plant material or bare soil were not significant for any predator group.

Invertebrate predation rates during the early milky ripening stage (BBCH code 73, late February) had an average of 31.1% per day (*SD* = 10.4% per day, *n* = 11), significantly higher than at any other phases (Tukey's *t* test, *p* < .001 for all comparisons). Moreover, invertebrate predation during the milky ripening stage (mean = 19.1% per day, *SD* = 7.6% per day, *n* = 11) and at cob ripening (mean = 15.2% per day, *SD* = 5.4% per day, *n* = 11) was significantly higher (Tukey's *t* test, *p* < .05 for both) than during late January (BBCH code 17–18) (mean = 8.6% per day, *SD* = 4.5% per day, *n* = 11). Ant predation was significantly (Tukey's *t* test, *p* < .01) higher in early January (BBCH code 15–16) (mean = 7.7% per day, *SD* = 3.4% per day, *n* = 11) than in late January, and marginally significantly higher (Tukey's *t* test, < .1) than during milky ripening stage (BBCH code 77) (mean = 3.4% per day, *SD* = 2.0% per day, *n* = 11) and at maize flowering (BBCH code 67) (mean = 5.8% per day, *SD* = 4.2% per day, *n* = 11). Predation by chewing insects was also significantly (Tukey's *t* test, *p* < .001) higher at the early milky ripening stage (mean = 27.4% per day, *SD* = 10.6% per day, *n* = 11) than other phenological phases.

Vertebrate predation peaked during ripening at 14.0% per day (*SD *= 10.0% per day, *n* = 11). This was significantly higher than any of the other sampling occasion (Tukey's *t* test, *p* < .05), except during early January (Tukey's *t* test, *p* < .1), and during milky ripening. Bird predation was significantly higher (Tukey's *t* test, *p* < .001–.05) at cob ripening (BBCH code 89) (mean = 11.5% per day, *SD* = 9.3% per day, *n* = 11) than at other times. Mammal predation peaked at maize flowering (mean = 3.9% per day, *SD* = 2.6% per day, *n* = 11).

## 4 DISCUSSION

Overall, we registered high predation pressure on the artificial caterpillars: nearly half of them were attacked within 24 hr in chaco serrano forest fragments. This is among the higher values recorded so far worldwide (Lövei & Ferrante, 2017). There are few data from cultivated fields (but see Howe, Nachman, & Lövei, 2015; Barbaro et al., 2017), and no published studies from maize fields from anywhere, making direct comparisons impossible. The ground level predation rate on artificial caterpillars found here was lower than in winter wheat in Denmark, and unsurprisingly, the relative contribution of the predatory groups responsible for the attacks was different: bird and ant predation rates were much higher in Argentina than in Denmark (Mansion‐Vaquié, Ferrante, Cook, Pell, & Lövei, 2017). These differences exist possibly due to the positive effect of landscape heterogeneity on farmland birds (Smith, Dänhardt, Lindström, & Rundlöf, 2010), and to the great ant abundance in subtropical areas (Hölldobler & Wilson, 1990), respectively.

Total predation, as well as predation by invertebrates, ants, chewing insects, and mammals were higher in forest fragments than in the crop, supporting our hypothesis 1. The same was not true for vertebrate and bird predation rates. It is plausible that habitat complexity plays a role for invertebrate predation rates, similar to invertebrate predator abundance (Langellotto & Denno, 2004). The difference in bird and mammal predation rates could result because dense vegetation makes these habitats less accessible for birds, while more attractive for mammals as they have a lower predation risk in habitats with taller vegetation (Doherty, Davis, & van Etten, 2015). The estimated predation pressure for all predators (except birds) was higher along edges than within the crop, but never significantly higher than within the forest fragments (H2 rejected). This indicates that forest fragment‐living predators regularly visited the edge, or that the edge supported a suit of predators that exerted predation pressure similar to the inner parts of a forest fragment. In this landscape, habitat complexity may be more important than complementarity of resources, because in addition to prey, invertebrate predators need favorable microclimatic conditions, and refuges (Langellotto & Denno, 2004).

Bigger fragments had higher predation rates by mammals, but lower ones by chewing insects (H3 partially supported). Negative relationships between invertebrate abundance and habitat area have been previously reported for a coccinellid predator (Elliott, Kieckhefer, & Beck, 2002) and ground‐dwelling insect predators were more abundant in small than big patches of chaco serrano (Moreno, Fernández, Molina, & Valladares, 2013). This becomes interpretable if we consider that small mammals are also predators of carabids, spiders, and other chewing insects. Small mammals may plausibly need bigger fragments to sustain populations where they exert a higher predation pressure on invertebrate prey—so chewing insects will, by corollary, become less abundant there than in smaller fragments. Such antagonism between carabids and small mammals is documented (Lövei & Sunderland, 1996) and was experimentally proven in semi‐arid habitats in North America (Parmenter & MacMahon, 1988).

Small mammal predation decreased as distance from the assumed closest source area increased, but the opposite was found for predation by birds. Difference in mobility may explain why mammal but not bird predation rates were so affected by relatively short distances.

Invertebrate and chewing insect predation rates increased with increasing distance from the continuous forest which was probably scale dependent. The mean distance of our fragments to the continuous source forest was 4.5 km, which may be too far to allow for regular movements between source and fragments for these invertebrates. These fragments have possibly become an “independent set of islands” with their own dynamics, and they no longer depend on the source. The opposite pattern was found for vertebrate predation rates, which suggests that vertebrate predators depended on these areas. Large and continuous forests frequently sustain large populations (Andrén, 1994; Pardini, de Souza, Braga‐Neto, & Metzger, 2005; Uezu, Metzger, & Vielliard, 2005) and can therefore be sources of individuals for nearby patches. Birds in the chaco serrano move actively between fragments (Díaz Vélez, Silva, Pizo, & Galetto, 2015). Moreover, the effect of isolation is highly influenced by the quality of the matrix (Prugh, Hodges, Sinclair, & Brashares, 2008). In our case, an important factor could be the low level of disturbance in this cultivation system: the maize fields in this area are very big, and between sowing and harvest, are very rarely disturbed, and this encourages the movement of birds.

We found no positive effect of the proximity to the forest edge to invertebrate predation (H4 rejected). This suggests that there was little spillover of invertebrate predators from the forest or only a part of the predators present in the forest edge moved into the matrix (Duelli & Obrist, 2003). Therefore, these forest fragments can act as sources of flying natural enemies to adjacent crops (González, Salvo, & Valladares, 2017b; González et al., 2015), but not of ground‐dwelling predators.

We found a positive correlation between total and chewing insect predation rates and live plant ground cover (H5 supported). Ground cover is important for soil surface‐active arthropods (Magura, 2002), which are probably a key group attacking artificial caterpillars (Ferrante et al., 2014; Mansion‐Vaquié et al., 2017), and which are abundant in chaco serrano (E. González, personal observation). Plant material affects soil pH, humidity, and organic content (Sadler, Small, Fiszpan, Telfer, & Niemela, 2006), and it is usually preferred by invertebrates to bare ground.

Crop phenology was an important factor for both invertebrate and vertebrate predators. Total predation, invertebrate, and chewing insect predation rates were higher during the early milky ripening stage, vertebrate, and bird predation rates reached their peak at cob ripening, and mammal predation rate was highest at maize flowering (H6 partially supported). The invertebrate predation rate peak may be explained by omnivorous predators using the most abundant resource available (i.e., pollen during flowering and prey after the end of it), as observed with the coccinellid *Coleomegilla maculata* in maize crops (Lundgren, Razzak, & Wiedenmann, 2004). Moreover, predatory insects not consuming pollen may show a delay, being attracted to the crop only when the prey density is already high (Evans, 1976), in our case at the phenological stage immediately after flowering. Small mammals may respond to an increase in prey faster than invertebrate predators because they move more (Brunner et al., 2013).

This study was the first application of the artificial caterpillars in Argentina, as well as in the chaco serrano. The scales at which different predators perceive the landscape, the relative permeability of the matrix, and predator mobility, can explain the patterns we observed. Landscape heterogeneity does not only support biodiversity in agro‐environments (Benton, Vickery, & Wilson, 2003), but also ESs such as biological control. The relationship between biodiversity and ESs is complex and can be described by various possible models (Tscharntke et al., 2005). Using direct measurements of ecosystem functioning, rather than “estimating” them by indirect measures, would help to articulate this complex relationship (Meyer, Koch, & Weisser, 2015). Both invertebrate and vertebrate predation rates can be affected by the same factor in different ways. The use of artificial caterpillars, which allows partitioning total predation rates to various predator groups, seems particularly suitable in such cases, and we encourage their wider use to understand factors influencing predation pressure in various habitats.

## CONFLICT OF INTEREST

None declared.

## AUTHOR CONTRIBUTIONS

MF, EG, and GL designed the study, field work was performed by MF and EG, data analysis and writing shared by all authors.
