# Supplementary material for: Predators do not spill over from forest fragments to maize fields in a landscape mosaic in central Argentina
Source: Ecol Evol. 2017 Aug 22;7(19):7699–707. doi: 10.1002/ece3.3247 (PMC5632606; doi:10.1002/ece3.3247)
Supplement: Supplementary file 1 [file ECE3-7-7699-s001.docx]

**Supplementary material to the article Predators do not spill over from forest fragments to maize fields in a landscape mosaic in central Argentina by** MARCO FERRANTE, Ezequiel GONZÁLEZ, GABOR L. LÖVEI


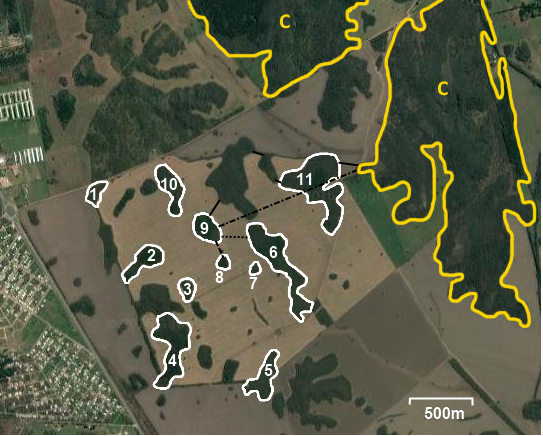


Fig.S1- Map of the study sites at Rio Ceballos, Córdoba, Argentina. The four measures of isolation are illustrated for fragment no. 9. Slashed line marks the shortest distance from the nearest neighbouring forest fragment (“Isolation 1”, in this case to fragment no. 8); a dotted line shows the shortest distance from the sampled edge of a fragment to the next one (“Isolation 2”; here to fragment no. 6); slash/dotted line marks the shortest linear distance to the nearest edge of the continuous forest (“Isolation 3”); solid lines show the shortest “stepping stone” distances to the continuous forest (“Isolation 4”). The continuous forest (C) is delineated in yellow.

**Table S1**. Landscape variables for each of the experimental forest fragment sites at Rio Ceballos, Córdoba, Argentina, during the southern summer of 2015/2016.

| **Site** | **Area (ha)** | **Perimeter (m)** | **Edge density** | **Isolation1 (m)** | **Isolation2 (m)** | **Isolation3 (m)** | **Isolation4 (m)** |
| --- | --- | --- | --- | --- | --- | --- | --- |
| 1 | 1.6 | 605 | 378.1 | 10 | 163 | 1450 | 594 |
| 2 | 3.9 | 1014 | 260.0 | 93 | 162 | 1510 | 633 |
| 3 | 1.0 | 439 | 439.0 | 26 | 280 | 1650 | 704 |
| 4 | 8.9 | 1789 | 201.0 | 87 | 168 | 1880 | 764 |
| 5 | 4.8 | 1208 | 251.7 | 24 | 202 | 1430 | 524 |
| 6 | 12.0 | 2483 | 206.9 | 16 | 196 | 832 | 199 |
| 7 | 0.5 | 273 | 546.0 | 66 | 212 | 1110 | 261 |
| 8 | 0.7 | 335 | 478.6 | 120 | 250 | 1280 | 427 |
| 9 | 3.6 | 741 | 205.8 | 122 | 234 | 1140 | 322 |
| 10 | 4.1 | 1050 | 256.1 | 25 | 289 | 999 | 359 |
| 11 | 15 | 2485 | 165.7 | 19 | 80 | 144 | 144 |

**Table S2.** Output of the final averaged models for predation rates on artificial caterpillars by all predators, and for each identified predator group at Rio Ceballos, Córdoba, Argentina, during the southern summer of 2015/2016**.** Factors under individual predator groups are ranked by their *z* values.

|  | **Estimated value** | **S.E.** | **Adjusted S.E.** | ***z* value** | **Significance, *p*** |
| --- | --- | --- | --- | --- | --- |
| *Total predation* |  |  |  |  |  |
| (Intercept) | -1.175 | 0.491 | 0.491 | 2.395 | 0.017 |
| Live Plant Cover | 2.024 | 0.203 | 0.203 | 9.973 | <0.0001 |
| Distance: 10m | -1.090 | 0.183 | 0.183 | 5.946 | <0.0001 |
| Distance: 2m | -1.030 | 0.181 | 0.181 | 5.681 | <0.0001 |
| Distance: 20m | -1.010 | 0.180 | 0.180 | 5.613 | <0.0001 |
| Distance: 1m | -0.981 | 0.179 | 0.180 | 5.464 | <0.0001 |
| Distance: 5m | -0.914 | 0.177 | 0.177 | 5.150 | <0.0001 |
| Phenology: Early Milky | 0.826 | 0.161 | 0.162 | 5.112 | <0.0001 |
| Distance: 40m | -0.837 | 0.176 | 0.176 | 4.749 | <0.0001 |
| Phenology: Ripe | 0.419 | 0.166 | 0.166 | 2.528 | 0.016 |
| Isolation1 | 2.767 | 1.112 | 1.113 | 2.488 | 0.013 |
| Phenology: BBCH17-18 | -0.435 | 0.184 | 0.184 | 2.369 | 0.018 |
| Phenology: Flowering | 0.380 | 0.166 | 0.166 | 2.290 | 0.022 |
| Phenology: Milky | 0.362 | 0.166 | 0.166 | 2.181 | 0.029 |
| Distance: Edge | 0.131 | 0.162 | 0.162 | 0.807 | 0.420 |
| *Predation by all invertebrates* | |  |  |  |  |
| (Intercept) | -1.581 | 0.256 | n.a. | -6.176 | <0.0001 |
| Phenology: Early Milky | 1.158 | 0.186 | n.a. | 6.216 | <0.0001 |
| Isolation 4 | 0.483 | 0.133 | n.a. | 3.623 | 0.0002 |
| Phenology: Flowering | 0.480 | 0.196 | n.a. | 2.446 | 0.014 |
| Phenology: Milky | 0.441 | 0.197 | n.a. | 2.241 | 0.025 |
| Phenology: Ripe | 0.153 | 0.204 | n.a. | 0.749 | 0.454 |
| Distance: Edge | -0.046 | 0.172 | n.a. | -0.268 | 0.789 |
| Phenology: BBCH17-18 | -0.522 | 0.229 | n.a. | -2.275 | 0.023 |
| Distance: 5m | -1.083 | 0.198 | n.a. | -5.484 | <0.0001 |
| Distance: 40m | -1.211 | 0.204 | n.a. | -5.932 | <0.0001 |
| Distance: 1m | -1.228 | 0.204 | n.a. | -6.020 | <0.0001 |
| Distance: 10m | -1.453 | 0.215 | n.a. | -6.743 | <0.0001 |
| Distance: 2m | -1.549 | 0.221 | n.a. | -7.015 | <0.0001 |
| Distance: 20m | -1.637 | 0.225 | n.a. | -7.285 | <0.0001 |
| *Predation by chewing insects* | |  |  |  |  |
| (Intercept) | -3.258 | 0.429 | 0.429 | 7.595 | <0.0001 |
| Live Plant Cover | 2.138 | 0.244 | 0.244 | 8.768 | <0.0001 |
| Phenology: Early Milky | 1.786 | 0.235 | 0.235 | 7.595 | <0.0001 |
| Phenology: Milky | 1.029 | 0.247 | 0.247 | 4.164 | <0.0001 |
| Phenology: Flowering | 0.919 | 0.250 | 0.250 | 3.680 | 0.0002 |
| Isolation 4 | 0.403 | 0.145 | 0.145 | 2.785 | 0.005 |
| Area | -0.036 | 0.014 | 0.014 | 2.555 | 0.011 |
| Perimeter | -0.191 | 0.088 | 0.088 | 2.164 | 0.030 |
| Phenology: Ripe | 0.517 | 0.262 | 0.262 | 1.969 | 0.049 |
| Phenology: BBCH17-18 | 0.031 | 0.284 | 0.284 | 0.109 | 0.913 |
| *Predation by ants* |  |  |  |  |  |
| (Intercept) | -1.324 | 0.399 | 0.399 | 3.321 | 0.0008 |
| Distance: 20m | -2.346 | 0.440 | 0.440 | 5.328 | <0.0001 |
| Distance: 2m | -2.032 | 0.390 | 0.390 | 5.207 | <0.0001 |
| Distance: 5m | -1.923 | 0.372 | 0.372 | 5.169 | <0.0001 |
| Distance: 1m | -1.813 | 0.357 | 0.357 | 5.080 | <0.0001 |
| Distance: 10m | -3.033 | 0.600 | 0.600 | 5.053 | <0.0001 |
| Distance: 40m | -1.685 | 0.344 | 0.344 | 4.899 | <0.0001 |
| Distance: Edge | -0.979 | 0.270 | 0.270 | 3.624 | 0.0003 |
| Phenology: BBCH17-18 | -1.304 | 0.380 | 0.380 | 3.430 | 0.0006 |
| Phenology: Milky | -0.870 | 0.334 | 0.334 | 2.606 | 0.009 |
| Phenology: Early Milky | -0.597 | 0.312 | 0.312 | 1.914 | 0.056 |
| Isolation1 | 4.277 | 2.54 | 2.541 | 1.683 | 0.092 |
| Phenology: Ripe | -0.444 | 0.300 | 0.300 | 1.479 | 0.139 |
| Isolation 4 | 0.303 | 0.284 | 0.284 | 1.067 | 0.286 |
| Phenology: Flowering | -0.299 | 0.291 | 0.291 | 1.026 | 0.305 |
| Isolation 2 | 2.130 | 2.146 | 2.147 | 0.992 | 0.321 |
| Area | -0.022 | 0.027 | 0.027 | 0.808 | 0.419 |
| *Predation by all vertebrates* | |  |  |  |  |
| (Intercept) | -1.871 | 0.275 | 0.275 | 6.814 | <0.0001 |
| Isolation 4 | -0.774 | 0.166 | 0.166 | 4.658 | <0.0001 |
| Isolation 1 | 5.890 | 1.755 | 1.756 | 3.355 | 0.0007 |
| Phenology: Ripe | 0.644 | 0.240 | 0.240 | 2.686 | 0.007 |
| Phenology: Early Milky | -0.190 | 0.275 | 0.275 | 0.691 | 0.490 |
| Phenology: Milky | 0.155 | 0.257 | 0.257 | 0.603 | 0.546 |
| Phenology: 7-8 | -0.098 | 0.269 | 0.269 | 0.365 | 0.715 |
| Phenology: Flowering | -0.022 | 0.265 | 0.266 | 0.082 | 0.935 |
| *Predation by birds* |  |  |  |  |  |
| (Intercept) | -4.581 | 0.608 | 0.608 | 7.538 | <0.0001 |
| Habitat: Maize | 1.708 | 0.460 | 0.460 | 3.716 | 0.0002 |
| Phenology: Ripe | 0.912 | 0.279 | 0.279 | 3.264 | 0.001 |
| Isolation 1 | 5.974 | 2.158 | 2.159 | 2.768 | 0.005 |
| Habitat: Forest | -1.632 | 10.980 | 10.980 | 1.487 | 0.137 |
| Isolation 4 | -0.376 | 0.261 | 0.261 | 1.439 | 0.150 |
| Phenology: Milky | 0.398 | 0.298 | 0.299 | 1.335 | 0.182 |
| Phenology: Flowering | -0.280 | 0.341 | 0.341 | 0.819 | 0.413 |
| Phenology: Early Milky | -0.226 | 0.336 | 0.337 | 0.673 | 0.501 |
| Phenology: BBCH17-18 | -0.124 | 0.328 | 0.328 | 0.377 | 0.706 |
| *Predation by mammals* | |  |  |  |  |
| (Intercept) | -2.452 | 1.040 | 1.040 | 2.358 | 0.018 |
| Habitat: Maize | -2.784 | 0.328 | 0.328 | 8.482 | <0.0001 |
| Area | 0.118 | 0.025 | 0.025 | 4.778 | <0.0001 |
| Isolation 2 | -10.410 | 2.199 | 2.199 | 4.732 | <0.0001 |
| Habitat: Forest | -0.513 | 0.296 | 0.296 | 1.736 | 0.083 |
| Phenology: Flowering | 0.401 | 0.436 | 0.437 | 0.918 | 0.359 |
| Phenology: Milky | -0.438 | 0.508 | 0.508 | 0.862 | 0.389 |
| Phenology: Ripe | -0.079 | 0.473 | 0.473 | 0.167 | 0.867 |
| Phenology: Early Milky | -0.070 | 0.473 | 0.473 | 0.148 | 0.882 |
| Phenology: BBCH17-18 | 0.003 | 0.464 | 0.465 | 0.005 | 0.996 |
